# Giant Omental Lymphangioma Presenting With Abdominal Pain and Intra-Cystic Hemorrhage in an Adult: A Case Report

**DOI:** 10.7759/cureus.113140

**Published:** 2026-07-22

**Authors:** Ryotaro Hashizume, Hiroshi Imai, Akinobu Hayashi, Tomomi Tanigawa, Fumito Ito, Naoya Noda, Atsushi Okamoto, Yasumitsu Kanamori, Mamiko Hamaji

**Affiliations:** 1 Department of Pathology and Matrix Biology, Mie University, Tsu, JPN; 2 Department of Genomic Medicine, Mie University Hospital, Tsu, JPN; 3 Pathology Division/Department of Genomic Medicine, Mie University Hospital, Tsu, JPN; 4 Pathology Division, Mie University Hospital, Tsu, JPN; 5 Department of Surgery, Ise Municipal General Hospital, Ise, JPN

**Keywords:** calretinin, case report, d2-40, greater omentum, immunohistochemistry, intra-abdominal cystic tumor, intra-cystic hemorrhage, omental lymphangioma

## Abstract

Lymphangiomas are benign tumors of lymphatic origin that usually arise in the neck and axilla during childhood; intra-abdominal involvement is rare, and an omental origin in an adult is exceptional. Because imaging features are non-specific, preoperative diagnosis is difficult, and intra-cystic hemorrhage can transform an otherwise indolent lesion into a surgical emergency. We report the case of a 46-year-old man who presented with sudden right lower-quadrant abdominal pain and a soft, mobile, tender right-abdominal mass. Contrast-enhanced computed tomography (CT) and magnetic resonance imaging (MRI) revealed a giant (approximately 25 cm), thin-walled, multilocular cystic mass containing a hematoma, with enhancement of the cyst walls but not the internal component, and feeding vessels arising from the greater omentum. Tumor markers were normal apart from mildly elevated carbohydrate antigen (CA) 125. Because malignancy could not be excluded, open resection was performed; the tumor arose from the greater omentum without invading adjacent organs and was completely excised. Histopathology confirmed an omental lymphangioma. The acute presentation was attributable to intra-cystic hemorrhage with a focal, self-sealed rupture superimposed on an indolent lesion. Because cross-sectional imaging cannot reliably distinguish lymphangioma from other cystic lesions, an immunohistochemical panel combining lymphatic and vascular endothelial markers (D2-40, CD31) with mesothelial markers (calretinin, WT-1) was decisive in excluding a mesothelial-lined cyst and mesothelioma. Omental lymphangioma should be considered in the differential diagnosis of a giant intra-abdominal multilocular cystic mass in adults, including those presenting acutely, and complete excision is both diagnostic and curative.

## Introduction

Lymphangiomas are benign tumors of the lymphatic system that arise from the localized proliferation of well-differentiated lymphatic channels. Approximately 95% occur in the head, neck, and axilla, and most are diagnosed in infancy or childhood. Intra-abdominal lymphangiomas comprise a small proportion of cases [1,2]. Among intra-abdominal sites, the mesentery is most frequently involved, whereas an omental origin in an adult is uncommon [2]. The occurrence of lymphangioma in adults is exceptional, with a reported incidence of approximately one in 20,000 to one in 250,000 hospital admissions [3].

The pathogenesis remains debated. Congenital maldevelopment of the lymphatic channels is considered the principal mechanism [1], but an acquired origin, in which trauma, inflammation, or previous surgery precipitates obstruction or stasis of lymphatic flow, has also been proposed [3,4]. Because the clinical and imaging features overlap with those of other cystic abdominal lesions, an accurate preoperative diagnosis is difficult, and the definitive diagnosis requires histopathological and immunohistochemical examination of the resected specimen [3,5]. Although intra-abdominal lymphangiomas in adults typically follow an indolent course, they can present acutely as a surgical emergency owing to intra-cystic hemorrhage, torsion, rupture, or infection [3,5].

We present this case to provide readers with a reproducible diagnostic approach to a giant multilocular cystic abdominal mass presenting acutely in an adult. The specific contribution of the present report is threefold. First, it documents the uncommon combination of a giant, omental-origin lymphangioma and an acute abdomen caused by intra-cystic hemorrhage with a focal, self-sealed rupture in an adult, a mechanism that is rarely captured with concordant imaging, operative, and pathological correlation. Second, it demonstrates the practical value of identifying omental feeding vessels on contrast-enhanced imaging for localizing the tumor origin preoperatively. Third, it emphasizes a focused immunohistochemical strategy, pairing lymphatic and vascular endothelial markers (D2-40, CD31) with mesothelial markers (calretinin, WT-1), that reliably distinguishes lymphangioma from its closest histological mimic, the mesothelial-lined cyst. Because cross-sectional imaging cannot reliably separate lymphangioma from other cystic abdominal lesions, including simple (mesothelial) mesenteric or omental cysts, mucinous cystic neoplasms, and cystic mesothelioma, the distinction rests on histopathology and immunohistochemistry; among lymphatic markers, D2-40 (podoplanin) is a highly specific marker of lymphatic endothelium and is therefore central to establishing the diagnosis.

This case was previously presented, in part, by the surgical team at the 323rd Ise Hospital-Clinic Liaison Case Conference (a local case conference, Ise, Japan) on May 25, 2026, and at the 304th Mie Surgical Society Meeting (a regional surgical meeting, Mie, Japan) on June 27, 2026.

## Case presentation

A 46-year-old man presented to a local clinic with sudden-onset, severe right lower-quadrant abdominal pain that started the previous night and prevented him from sleeping. He was referred to our hospital the same day. Past history included right inguinal hernia repair in childhood and laparoscopic cholecystectomy approximately 10 years before presentation, and blunt abdominal trauma in adolescence. He was a former smoker (14 pack-years) who had recently quit and did not drink alcohol. He worked as a care worker, took no regular medications, and reported no allergies. His family history was unremarkable.

Although advised to be hospitalized for further evaluation, he elected to return home because there was resolution of his pain and some personal reasons, and admission was arranged for the next available weekday. He was admitted two days later. Physical examination revealed a height of 183.5 cm, weight of 72.2 kg, and body mass index of 21.5 kg/m2. The right abdomen was mildly distended, and a soft, mobile, tender mass without rebound tenderness was palpable in the right lower quadrant. Laboratory tests showed a mildly elevated white blood cell count, C-reactive protein, and lactate dehydrogenase, a marginally low hemoglobin, and mildly elevated carbohydrate antigen (CA) 125; the remaining blood counts, biochemistry, coagulation parameters, and tumor markers were within normal limits (Table 1).

**Table 1:** Laboratory data on admission Values outside the institutional reference range are indicated as elevated or low.

Parameter	Value	Reference range
White blood cells	9,700/µL (elevated)	3,300–8,600/µL
Hemoglobin	13.3 g/dL (low)	13.7–16.8 g/dL
Platelets	258 × 10³/µL	158–348 × 10³/µL
Total protein	6.7 g/dL	6.6–8.1 g/dL
Albumin	4.1 g/dL	4.1–5.1 g/dL
Total bilirubin	0.9 mg/dL	0.4–1.5 mg/dL
Aspartate aminotransferase	20 U/L	13–30 U/L
Alanine aminotransferase	15 U/L	10–35 U/L
Lactate dehydrogenase	233 U/L (elevated)	124–222 U/L
Blood urea nitrogen	12.1 mg/dL	8–20 mg/dL
Creatinine	0.66 mg/dL	0.65–1.07 mg/dL
C-reactive protein	2.46 mg/dL (elevated)	0–0.14 mg/dL
Prothrombin time	95%	70–130%
Activated partial thromboplastin time	31.9 s	24–34 s
Carcinoembryonic antigen	0.9 ng/mL	≤5 ng/mL
Carbohydrate antigen 19-9	3.3 U/mL	≤37 U/mL
Carbohydrate antigen 125	35.2 U/mL (elevated)	≤35 U/mL
Soluble interleukin-2 receptor	401 U/mL	204–587 U/mL

Imaging tests were carried out on the day of admission. Abdominal ultrasonography showed a multilocular cystic mass (Figure 1a), and color Doppler demonstrated almost no internal blood flow (Figure 1b). 

**Figure 1:**
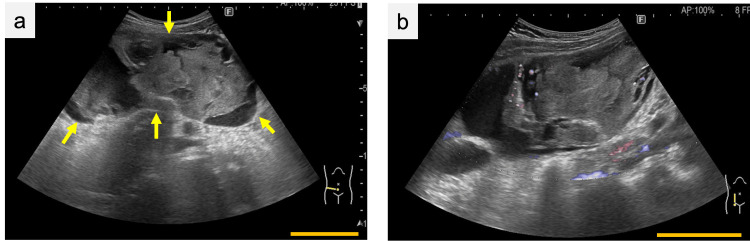
Preoperative abdominal ultrasonography (a) B-mode ultrasonography shows a multilocular cystic mass (arrows). (b) Color Doppler ultrasonography demonstrates almost no internal blood flow. Scale bars: 5 cm.

Computed tomography (CT) revealed a thin-walled, multilocular cystic mass in the right abdomen containing high-attenuation material consistent with a hematoma on unenhanced images (Figure 2a). After contrast administration, the cyst walls enhanced but the internal component did not (Figure 2b), and coronal reconstruction demonstrated the craniocaudal extent of the mass (Figure 2c). Vessels arising from the greater omentum supplied the mass, and there was no continuity with the appendix and no contrast extravasation; a small amount of ascites was present.

**Figure 2:**
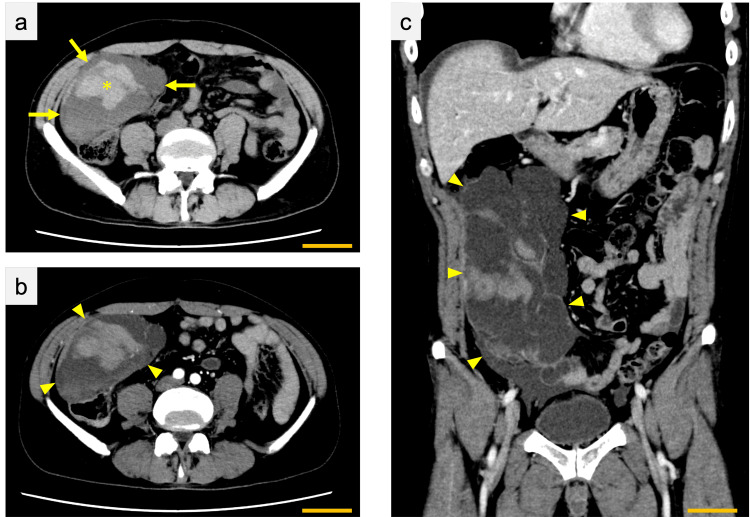
Preoperative CT of the giant intra-abdominal mass (a) Unenhanced axial CT shows a thin-walled, multilocular cystic mass occupying the right abdomen (arrows) containing high-attenuation material (asterisk) consistent with a hematoma. (b) Contrast-enhanced axial CT shows enhancement of the thin cyst walls (arrowheads) without enhancement of the internal high-attenuation component; no contrast extravasation was identified. (c) Coronal reconstruction demonstrates the craniocaudal extent of the mass (arrowheads). Scale bars: 5 cm. CT: computed tomography

Magnetic resonance imaging (MRI) demonstrated a multilocular cystic mass containing a hematoma, showing low signal intensity on T1-weighted imaging with an internal high-signal area, and high signal intensity on T2-weighted imaging with an internal low-signal area, alongside mild enhancement and thickening of the wall; malignancy could not be excluded (Figure 3). Colonoscopy revealed no mucosal lesion and a normal appendiceal orifice.

**Figure 3:**
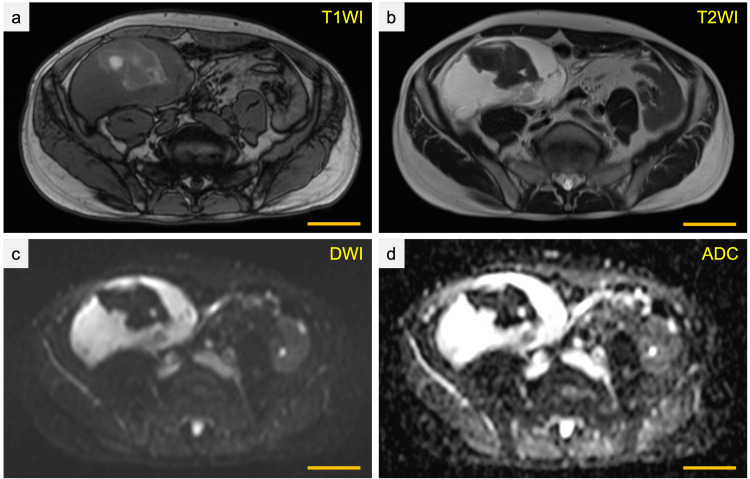
Preoperative magnetic resonance imaging of the cystic mass Axial images were obtained at the same level. (a) On T1-weighted imaging, the mass shows low signal intensity with an internal high-signal area, indicating a hematoma. (b) On T2-weighted imaging, the mass shows high signal intensity with an internal low-signal area. (c) On diffusion-weighted imaging, the lesion shows high signal intensity with an internal low-signal area, with the corresponding (d) apparent diffusion coefficient map. Scale bars: 5 cm.

The preoperative diagnosis was a giant intra-abdominal cystic tumor, most likely of omental origin (approximately 25 × 7 cm). The differential diagnosis included mucinous cystic neoplasm and lymphangioma, as well as gastrointestinal stromal tumor, schwannoma, sarcoma, and liposarcoma. Because malignancy could not be excluded, open tumor resection was performed eight days after admission for diagnostic and therapeutic purposes. The patient experienced no abdominal pain from admission to the day of surgery.

Through an upper-to-lower midline incision, hemorrhagic ascites was found, and a multilocular cystic tumor arising from the greater omentum was identified. The tumor did not adhere to or invade the colon or mesentery, although it was focally adherent to the right lower anterior abdominal wall (Figure 4a). Gentle blunt dissection at this site disclosed a thinned, defective area of the cyst wall, from which dark-red hemorrhagic content of similar appearance to the peritoneal ascites was released. The feeding vessel branching from the left gastroepiploic vessel was ligated, and the tumor was completely excised without combined organ resection. The operative time was approximately 2.9 hours, and blood loss including ascites was 119 mL, with no transfusion required. The resected specimen was a 19.6 × 13.5 cm multilocular cystic mass with a partial hematoma (Figures 4b-4c).

**Figure 4:**
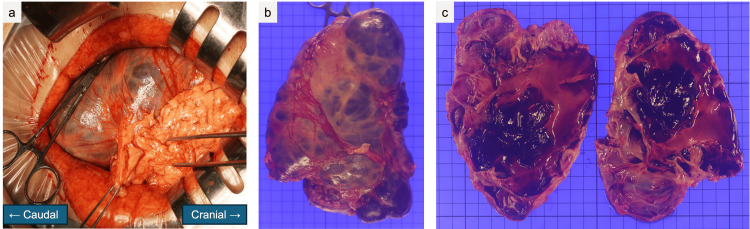
Intraoperative findings and the resected specimen (a) Intraoperative photograph showing the tumor arising from the greater omentum; no adhesion to the colon or mesentery was observed (cranial, right; caudal, left). (b) External surface of the resected specimen, a multilocular cystic mass measuring 19.6 × 13.5 cm. (c) Cut surface showing multiple cystic loculi containing a partial hematoma. Background grid squares in (b) and (c) each measure 1 cm; the intraoperative photograph (a) is not to scale.

Histopathologically, the lesion showed variably sized cystic spaces lined by a single layer of flat endothelial cells without cytological atypia (Figure 5). The lining cells were positive for D2-40 and CD31, focally positive for CD34, and negative for calretinin and WT-1, confirming a lymphangioma of the greater omentum (Figure 6). The postoperative course was uneventful, and the patient was discharged on postoperative day 9.

**Figure 5:**
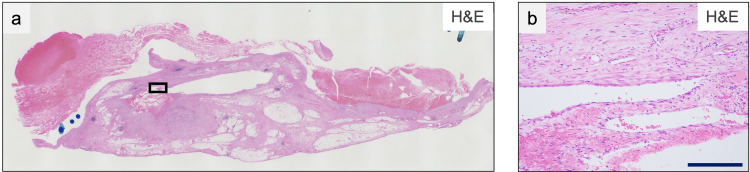
Histopathological findings (H&E) (a) Whole-mount montage (×4 objective). (b) Cystic space is lined by a single layer of thin, flat endothelial cells, consistent with lymphangioma (H&E, ×200) The boxed area in (a) is shown in (b). Scale bar: 200 µm in (b). H&E: hematoxylin and eosin

**Figure 6:**
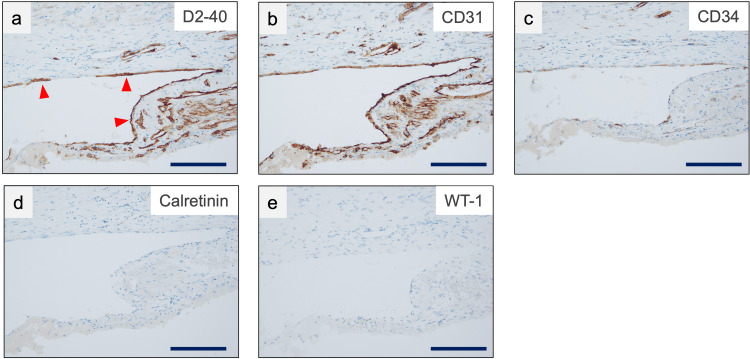
Immunohistochemical findings (a) D2-40, ×200: positive lining endothelial cells (red arrowheads). (b) CD31, ×200: positive. (c) CD34, ×200: focal, weak positivity in approximately 30% of the lining endothelial cells. (d) Calretinin, ×200: negative. (e) WT-1, ×200: negative. Scale bars: 200 µm.

At the first postoperative outpatient visit approximately one month after surgery, once the pathological diagnosis had been established, the patient was asymptomatic and in good general condition, with unremarkable laboratory findings; no imaging was obtained at that visit. He has remained asymptomatic thereafter, and surveillance non-contrast CT is planned at three months postoperatively to evaluate for recurrence.

## Discussion

Intra-abdominal lymphangiomas are rare, particularly of omental origin in adults [2,4], as in our case. Although abdominal lymphangiomas in adults tend to follow a longer, more indolent course than those in children [1], adult cases that present acutely or reach a giant size, occasionally requiring resection of adjacent organs, continue to be reported [4]. The present tumor was both giant (19.6 × 13.5 cm) and complicated by intra-cystic hemorrhage.

The cause of lymphangioma in adults is uncertain. In addition to congenital lymphatic maldevelopment, an acquired mechanism related to lymphatic obstruction after trauma, inflammation, or surgery has been suggested [3,4]. Our patient had undergone laparoscopic cholecystectomy approximately 10 years earlier, at which time no mass had been noted, and he reported blunt abdominal trauma in adolescence. These events raise the possibility of an acquired lesion, although this remains speculative.

Adults with intra-abdominal lymphangioma are frequently asymptomatic or report only non-specific complaints; however, complications such as intra-cystic hemorrhage, torsion, rupture, or infection can precipitate an acute abdomen [5,6]. In the present patient, the sudden onset of pain coincided with hemorrhage into the cyst. Imaging revealed high-attenuation, T1-hyperintense material within the locules consistent with a hematoma, and at surgery a focal adhesion to the right lower anterior abdominal wall overlaid a thinned, defective area of the cyst wall, from which dark-red hemorrhagic content identical in appearance to the peritoneal ascites was released. The matching appearance of the intra-cystic content and ascites, together with the localization of the wall defect at the site of adhesion, indicates that the cyst had bled and ruptured at this point, leading to extravasation into the peritoneal cavity. After the initial pain episode, the patient remained symptom-free from admission until surgery, consistent with a single discrete rupture that subsequently sealed via adhesion to the abdominal wall rather than an ongoing hemorrhagic process. Intra-cystic hemorrhage with focal, self-sealed rupture may therefore explain the acute presentation of an otherwise indolent lesion, although this mechanism remains inferential.

Although cross-sectional imaging typically reveals a thin-walled, multilocular cystic mass, this finding cannot reliably distinguish lymphangioma from other cystic abdominal lesions, including simple (mesothelial) mesenteric or omental cysts, cystic mesothelioma, and cystic neoplasms, necessitating histopathology [3,7]. In the present case, an epithelial neoplasm such as a mucinous cystic neoplasm was excluded because the locules were lined by a single layer of flat endothelial cells expressing D2-40 and CD31 rather than mucinous epithelium, whereas a mesothelial-lined cyst and cystic mesothelioma were excluded by the absence of calretinin and Wilms tumor 1 (WT-1) expression. In our patient, the intra-cystic hematoma and the mildly thickened, enhancing wall mimicked a solid or possibly malignant component. These findings, alongside mild elevation of CA125, a marker of limited specificity that can also be mildly elevated in benign lymphangioma [8], precluded the exclusion of malignancy before surgery and prompted complete open resection. Contrast-enhanced CT also demonstrated that the vessels supplying the mass arose from the greater omentum; identifying such feeding vessels can help localize the omental origin of these lesions [8].

The principal differential histological diagnosis of omental lymphangioma is a mesothelial-lined cyst, which may initially be mistaken for it [7]. Immunohistochemistry was therefore decisive: the lining cells were positive for the lymphatic and vascular endothelial markers D2-40, CD31, and (variably) CD34 and negative for the mesothelial markers calretinin and WT-1, in line with prior reports [6,7], confirming lymphangioma and excluding a mesothelial-lined cyst or mesothelioma.

Complete surgical excision is the treatment of choice, both to obtain a definitive diagnosis and to prevent recurrence, as incompletely resected lesions can recur [2,3]. When the tumor is adherent to or arises in continuity with adjacent organs, en bloc resection may be required [4], whereas a laparoscopic or laparoscopic-assisted approach can be appropriate for smaller, uncomplicated lesions; open resection is preferred for giant or complicated tumors [7,8]. For unresectable lesions, sclerotherapy has been used as an alternative [6]. In the present patient, the tumor arose from the greater omentum and was supplied by a branch of the left gastroepiploic artery; it was completely excised without resection of other organs, and the postoperative course was uneventful, consistent with the favorable prognosis reported after complete excision [2,3].

Strengths and limitations

A strength of this report is the concordant clinical, radiological, operative, and pathological documentation of an acute presentation caused by intra-cystic hemorrhage, together with a defined immunohistochemical panel of cross-specialty relevance. The main limitation is that a single case cannot establish the frequency of acquired mechanisms or the optimal surveillance interval, and the proposed acquired etiology remains speculative. In addition, postoperative follow-up is currently limited to approximately one month, with surveillance imaging (non-contrast CT) planned at three months, so that the long-term clinical course and recurrence risk remain uncertain. As with any single case report, generalizability is limited. Nonetheless, the key clinical lessons are that omental lymphangioma should be included in the differential diagnosis of a giant multilocular cystic abdominal mass in an adult even when the presentation is acute, that intra-cystic hemorrhage with focal rupture can mimic malignancy on imaging, and that a targeted immunohistochemical panel (D2-40 and CD31 positive; calretinin and WT-1 negative) is required to distinguish lymphangioma from a mesothelial-lined cyst and to guide complete, curative excision.

## Conclusions

Omental lymphangioma is a rare benign tumor of lymphatic origin. We encountered a giant omental lymphangioma in a 46-year-old man who presented with acute abdominal pain attributable to intra-cystic hemorrhage. Omental lymphangioma should be considered in the differential diagnosis of a giant multilocular cystic abdominal mass in adults, including those presenting as an acute abdomen. Because imaging is non-specific, the diagnosis rests on histopathology with immunohistochemistry; a panel combining lymphatic and vascular endothelial markers (D2-40, CD31, CD34) with mesothelial markers (calretinin, WT-1) is essential to distinguish lymphangioma from mesothelial-lined cysts. Complete surgical excision is curative and provides the definitive diagnosis.

## References

[1] de Perrot M, Rostan O, Morel P, Le Coultre C (1998). Abdominal lymphangioma in adults and children. Br J Surg.

[2] Rao TN, Parvathi T, Suvarchala A (2012). Omental lymphangioma in adults-rare presentation report of a case. Case Rep Surg.

[3] Mhand M, Rhoul C, Bouhout T, Serji B (2024). Cystic lymphangioma of the mesentery in an adult: a case report and literature review. Cureus.

[4] Aldhaheri RH, Alabdulaaly NI, Aldosari SH, Altoukhi KH, Alobaid SS (2025). Successful surgical management of omental lymphangioma in an adult: a case report. Cureus.

[5] Jha T, Sharma M, Ahuja A (2024). Mesenteric cystic lymphangioma in adults: a rare entity presenting as acute abdomen - a report of two cases. Autops Case Rep.

[6] Boujguenna I, Chiboub N, Boukis F, Abdouh S, Fakhri Y (2025). Mesenteric cystic lymphangioma in three adults. J Surg Case Rep.

[7] Aliukonis V, Lasinskas M, Pilvelis A, Gradauskas A (2021). Pathological discrepancy: simple mesenteric cyst vs. mesenteric lymphangioma. Case Rep Surg.

[8] Fujii T, Tanaka A, Katami H, Shimono R (2025). Utilizing contrast-enhanced computed tomography for accurate localization of an omental lymphangioma: a case report. Cureus.

